# Sustainable intensification of maize and wheat cropping system through pulse intercropping

**DOI:** 10.1038/s41598-021-98179-2

**Published:** 2021-09-22

**Authors:** S. C. Tripathi, Karnam Venkatesh, Raj Pal Meena, Subhash Chander, G. P. Singh

**Affiliations:** ICAR-Indian Institute of Wheat and Barley Research, Agarsain Marg, P.B. No. 158, Karnal, Haryana 132 001 India

**Keywords:** Plant sciences, Climate sciences

## Abstract

The intercropping of legumes with cereals help to achieve sustainable intensification by their mutual complementarity at efficiently using radiation, nutrients, etc. Several studies indicated such beneficial effects on the other component crop however, little research has been conducted to quantify their effects on the subsequent crop in a cropping system. In this study, the effect of the legume intercropping on the entire cropping system, particularly the maize + legume-wheat system was studied. Four legumes intercropped to maize followed by wheat crop were studied for intensification measures such as wheat equivalent yield (WEY), land equivalent ratio (LER), sustainable value index (SVI), and economic returns. N saving effect of legumes on the subsequent wheat crop was quantified with two N levels. Maize + cowpea-wheat combination was the most productive and economic intercrop combination (LER = 1.71, SVI = 0.96) with an increase in net economic return (43.63%) with a B:C ratio of 1.94. An additional 25% N (37.5 kg ha^−1^) was saved in the wheat crop when the legume intercropping was undertaken with maize. The results suggest that intercropping is the key to diversification and reduces the risk of crop failures by enhancing land-use efficiency, soil fertility, and economic returns under weather vagaries. This will be beneficial to small and marginal farmers of many countries.

## Introduction

The major goal of agriculture during the current times is sustainably increasing food production without harming the environment^1^. Intensive mono-cropping of cereals globally has contributed to increased production of staple crops but at the cost of disturbance to the ecological balance in terms of causing pollution, overexploitation of resources etc^2–4^. Latest innovations in agronomy such as intercropping have contributed to enhanced land utilization by efficiently choosing crop species with complementarity for space, radiation, and input usage^5^. Intercropping was proven to help in improving resource capture and utilization, soil fertility, and reduce soil erosion^6,7^. Two crops with different canopy coverage, architecture, and growing periods enhance radiation use efficiency by reducing the light reaching the ground^5^. Maize/soybean^5,8^, maize/cowpea^9,10^ and maize/peanut^11,12^ are the examples for such intercrops.

Globally, maize has a significant area under cultivation (197.23 mha) and out of which 73% is occupied by developing countries. Some of the countries like China (41.31 mha), the USA (32.95 mha), Brazil (17.52 mha), India (9.03 mha), Argentina (7.23 mha), South Africa (2.30 mha), and Ethiopia (2.27 mha), etc. have a great scope of intercropping owing to their high maize acreage and mechanized form of seeding^13^. Maize is preceded by wheat in many counties like China, India, Mexico, and Pakistan, etc. Maize constitutes 6.5% of the food supplies in Asia and thus is a major contributor to food security of the region^14^. Identification and development of agronomic manipulations and mechanisms such as cereal + pulse intercropping may enhance the sustainability of maize-based cropping systems worldwide. Maize/pulse intercropping can significantly reduce the competition for land resources between maize and legumes can help in simultaneously increasing the production of both crops^15^. Additional pulses produced through intercropping can contribute to overall enhanced pulse production. India is home to the largest pulse-consuming population globally^16^ and 20% of its demand is met by imports valued to the tune of 3800 USD in 2016–2017^17^.

Intercropping of pulses with maize was found to be advantageous to maize and however, little research has been conducted to quantify their effects on the subsequent crop in a cropping system. The reported favorable effects of the pulse intercropping with cereals include high carbon sequestration, higher water use efficiency, nitrogen transfer to the subsequent crop^18^, improve soil biodiversity etc^19^. Therefore, the study was designed to study the effect of the pulse intercropping with maize on system yield and quantification of the N saved in the subsequent wheat crop.

## Materials and methods

### Study site and soil characteristics

A field experiment was conducted during 2016–2017 to 2019–2020 at the research farm of ICAR-Indian Institute of Wheat and Barley Research, Karnal, Haryana, India (29°43′ N, 76°58′ E, 245 m above sea level). The weather during July to October remains hot and humid, and most of the precipitation (75–80%) occurs during this period. The soil was moderately well-drained coarse-textured sandy loam (11.1% clay, 26.5% silt, 62.4% sand) with low to moderate fertility. Baseline soil samples were collected (0–15 cm depth) from each test site at the start of the experiment (2016–2017) and after completion of four crop cycles and analyzed for pH (using a soil water solution of 1:2.5 wt/v), soil organic carbon^20^, available N^21^, available P^22^, and available K^23^. The soil was having 111.88 kg ha^−1^ available N, 0.35% organic carbon, 12.37 kg ha^−1^ available P, and 155.9 kg ha^−1^ available K with an alkaline pH of 8.44 and EC of 0.12 dS m^−1^.

### Meteorological conditions

**Figure 1:**
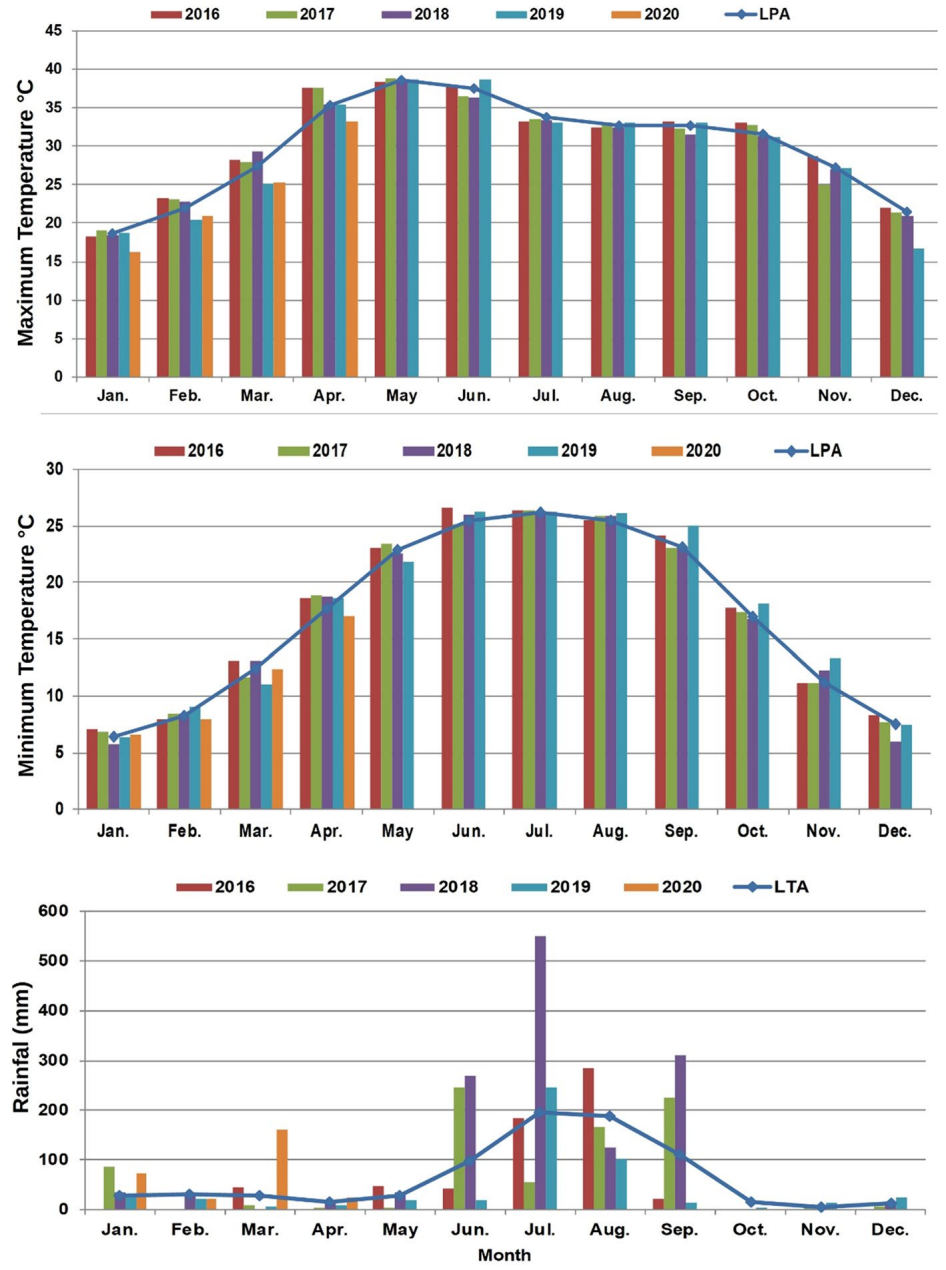
Maximum and minimum temperature and rainfall during the crop years.

The data on important weather parameters such as daily minimum, maximum temperature, and precipitation were recorded throughout the crop season during the experimental years. The long-term weather data from 1981 to 2018 were collected from the data repository maintained by ICAR- Central Soil Salinity Research Institute, Karnal. The monthly temperature (minimum and maximum) and precipitation data were used to calculate the long-term average values. The weather information is presented in the graph (Fig. 1). The highest precipitation was recorded in the year 2020, and the lowest was recorded in the year 2016. July month, starting monsoon month of the year received the maximum rainfall and particularly it was highest in 2019, even higher than the long term average. In 2019 and 2020, the maximum temperature from February to April was lower than that of other years whereas there was no difference in minimum temperature. In contrast, the first 2 years’ maximum and minimum temperature of March and April was higher than the long-term average.

### Experimental design, treatments and crop management

The experimental design adopted was a randomized block design with 3 replications and consisted of 13 treatments (Table 1). Four legumes i.e. green gram (variety SML 668), black gram (variety T-9), cluster bean (variety HG 365), and cowpea (variety PusaKomal) were used in this study, and cultivars were collected from the local market. Authors comply with the IUCN Policy Statement on Research Involving Species at Risk of Extinction and the Convention on the Trade in Endangered Species of Wild Fauna and Flora. Four legumes i.e. green gram, black gram, cluster bean, and cowpea were intercropped with maize crop in 1:1 ratio and a sole crop also was planted as control. During the summer season, maize, green gram, black gram, cluster bean, and cowpea were grown sole crop as well as intercrop. Maize variety X 92 (hybrid) with seed rate of 25 kg ha^−1^, green gram variety SML 668 with seed rate 20 kg ha^−1^, black gram variety T-9 with seed rate of 20 kg ha^−1^, cluster bean variety HG 365 with seed rate of 10 kg ha^−1^ and cowpea variety Pusa Komal with seed rate of 25 kg ha^−1^ were used. Wheat variety HD 3086 was seeded during the winter season with a seed rate of 100 kg ha^−1^. Wheat crop was grown in plots where previous crops was the sole legume, 112.5 kg N was applied. In maize crop a uniform fertilizer rate of 150 kg N, 60 kg P_2_O_5_ and 40 K_2_O kg ha^−1^ was applied to avoid any nutrient stress as per the recommended package of practices^24^. The whole quantity of phosphorus and potassium and one-third of N was applied as basal dose through urea, diammonium phosphate, and muriate of potash. The remaining N was applied as top dressing in two equal splits, DC31 and DC 41^25^. Treatments having sole legume crops were applied with 20 kg N ha^−1^ and 50 kg P_2_O_5_ ha^−1^. Surface irrigation was applied as per recommended scheduling at critical growth stages. Weeds in maize and legumes were controlled by practicing two intercultural operations with the help of a hand-held hoe. For weed control in wheat two herbicides were sprayed i.e. sulfosulfuron @ 25 g ha^−1^ and metsulfuron @ 4 g ha^−1^, respectively in 400 L of water at 30 days after sowing. A net plot of 14.4 m^2^ (maize) and 9.8 m^2^ (wheat) was harvested manually from the middle of the experimental plot leaving the border on all sides at physiological maturity with the help of sickles. All the other recommended package of practices were kept common in all the crops as per recommendation^24^. Yield and yield attributing characters were obtained by using methods as described by Bell and Fischer^26^.

**Table 1:** Treatment details of the long term irrigated maize + pulses-wheat intercropping system.

	Treatments	Crops grown	Annual fertilizer application (kg ha^−1^)	
			Maize/pulses	Wheat
1	MGW100	Maize + green gram − wheat	150 N, 60 P_2_O_5_, 40 K_2_0	150 N, 60 P_2_O_5_, 40 K_2_0
2	MGW75	Maize + green gram − wheat	150 N, 60 P_2_O_5_, 40 K_2_0	112.5 N, 60 P_2_O_5_, 40 K_2_0
3	MBW100	Maize + black gram − wheat	150 N, 60 P_2_O_5_, 40 K_2_0	150 N, 60 P_2_O_5_, 40 K_2_0
4	MBW75	Maize + black gram − wheat	150 N, 60 P_2_O_5_, 40 K_2_0	112.5 N, 60 P_2_O_5_, 40 K_2_0
5	MCW100	Maize + cluster bean − wheat	150 N, 60 P_2_O_5_, 40 K_2_0	150 N, 60 P_2_O_5_, 40 K_2_0
6	MCW75	Maize + cluster bean − wheat	150 N, 60 P_2_O_5_, 40 K_2_0	112.5 N, 60 P_2_O_5_, 40 K_2_0
7	MCowW100	Maize + cowpea − wheat	150 N, 60 P_2_O_5_, 40 K_2_0	150 N, 60 P_2_O_5_, 40 K_2_0
8	MCowW75	Maize + cowpea − wheat	150 N, 60 P_2_O_5_, 40 K_2_0	112.5 N, 60 P_2_O_5_, 40 K_2_0
9	MW	Maize − wheat	150 N, 60 P_2_O_5_, 40 K_2_0	150 N, 60 P_2_O_5_, 40 K_2_0
10	GW	Green gram − wheat	20 N, 50 P_2_O_5_	112.5 N, 60 P_2_O_5_, 40 K_2_0
11	BW	Black gram- wheat	20 N, 50 P_2_O_5_	112.5 N, 60 P_2_O_5_, 40 K_2_0
12	CW	Cluster bean − wheat	20 N, 50 P_2_O_5_	112.5 N, 60 P_2_O_5_, 40 K_2_0
13	CowW	Cowpea − wheat	20 N, 50 P_2_O_5_	112.5 N, 60 P_2_O_5_, 40 K_2_0

### Observations recorded

Grain yield was calculated from the net plot area and converted into kg ha^−1^. Grains of maize, green gram, and black gram were taken as yield whereas green pods of cluster bean and cowpea were considered as yield. In the case of wheat, HI was calculated by dividing grain yield by biomass. The number of earheads per meter row length was counted at two places in each plot and converted to per m^2^. Thousand grains weight (TGW) was calculated by taking random grain samples and counted by using Contador electronic seed counter (Pfeuffer, Germany) and weighed. Layak et al.^27^ used various formulae to calculate intercrop productivity and efficiency and accordingly these formulae were used in the present study to arrive at the distinct trend.

### System productivity

System productivity in terms of wheat equivalent yield WEY was calculated by multiplying yield with minimum support price/market price of each crop in a cropping sequence and subsequently adding and thereafter divided by the price of one-tonne wheat.

1 $$WEY= (Yield of intercrops x market price of intercrops/ Market price of wheat)$$

### Land equivalent ratio (LER)

LER is the relative area of the sole crop that would be required to produce the equivalent yield achieved by intercropping. It is the summation of the ratio of yield of intercrop to the yield of the sole crop. It was calculated as follows^28^:

2 $${\text{LER}} = {\text{LER}}^{{\text{M}}} + {\text{LER}}^{{\text{L}}} = \left( {{\text{Y}}^{{{\text{ML}}}} {\text{/Y}}^{{{\text{MM}}}} + {\text{Y}}^{{{\text{LM}}}} {\text{/Y}}^{{{\text{LL}}}} } \right)$$

where LER^M^ and LER^L^ are the partial LER of maize and legumes, respectively. Y^ML^ = Yield of first intercrop, Y^LM^ = Yield of second intercrop, Y^MM^ = Yield of first sole crop, Y^LL^ = Yield of second sole crop.Monetary Advantage Index (MAI)

The MAI is computed by using the following formula:

3 $${\text{MAI}} = \left\{ {\left( {{\text{LER}} - 1} \right)/\left( {{\text{LER}}} \right) \times {\text{ value}}\;{\text{ of }}\;{\text{combined }}\;{\text{intercrops}}} \right\}$$

### Sustainability value index (SVI)

4 $${\text{SVI}} = (\mu - \delta )/{\text{Y}}_{\max } ,$$

where, μ = mean of particular treatment in monetary terms, δ = standard deviation of particular treatment in monetary terms and Y_max_ = potential maximum monetary returns (by converting potential maximum yield in monetary terms) over the years. Sustainable value index was calculated as per procedure earlier reported^29^.

### Economics

Maize, green gram, black gram, cluster bean, cowpea, wheat crops yield was multiplied by minimum support price (US $187.5, 733.5, 710.5, 565.8, 131.6, 228.3 ton^−1^, respectively) of these crops (ref for MSP from PIB, GOI). The wheat straw yield was also multiplied by the market rate ($32.9 ton^−1^) and added to get the gross return. Cost of cultivation was calculated by considering field preparation, seed, fertilizer, irrigation, transportation, herbicide application, the cost involved in harvesting and threshing of produce, management charges, the rental value of land, interest on fixed capital, depreciation cost of implements, and farm buildings. Net return was calculated by subtracting the cost of cultivation from gross returns. The benefit–cost ratio was calculated by dividing the gross return by the total cost of cultivation. To convert into US $ gross return, cost of cultivation and net return were divided by prevailing exchange rate ($ = Rs 76).

### Statistical analysis

Analysis of variance (ANOVA) and ranking of treatments was completed using Tukey’s Range test at 0.05 (5%) level of significance. The General Linear Model (GLM) Procedure in SAS^®^9.3 version 6.1.7061 for Windows (Cary, NC, SAS Institute Inc., 2012) was used for statistical analysis.

## Results

### Analysis of variance

Intercropping treatments of various pulse crops with maize were having significant effect on all the traits recorded in wheat such as GY (P < 0.001), BM (P < 0.001), HI (P < 0.001), SNPMS (P = 0.045), TGW (P < 0.001), GPS (P = 0.017) and GrPMS (P < 0.001). The intercropping combinations were also having a statistically significant effect on overall monetary returns parameters such as NR and B:C ratio (Table 2).

**Table 2:** Analysis of variance of maize + pulse-wheat intercropping system.

Effect	df	GY	BM	HI	SNPMS	TGW	GPS	GrPMS	WEY	Returns ($)	Net returns ($)	B:C ratio
Year	3	< 0.001	< 0.001	< 0.001	< 0.001	< 0.001	< 0.001	< 0.001	< 0.001	< 0.001	< 0.001	< 0.001
Treat	12	< 0.001	< 0.001	< 0.001	0.045	< 0.001	0.017	< 0.001	< 0.001	< 0.001	< 0.001	< 0.001
Year × Treat	36	0.021	< 0.001	0.002	0.058	0.011	0.010	0.021	< 0.001	< 0.001	< 0.001	< 0.001

### Wheat yield and yield attributing parameters

Legume intercrops and sole crops were followed with wheat in the winter season at two levels of nitrogen fertility viz. recommended N rate (150 kg N ha^−1^) and 25% less than RDF (112.5 kg N ha^−1^). The parameters under study were GY, BM, GPS, TGW, SpPMS, HI, GrPMS were non-significant except HI. MCowW75 combination yielded more than maize–wheat cropping sequence besides saving 25% N.

### System productivity

**Figure 2:**
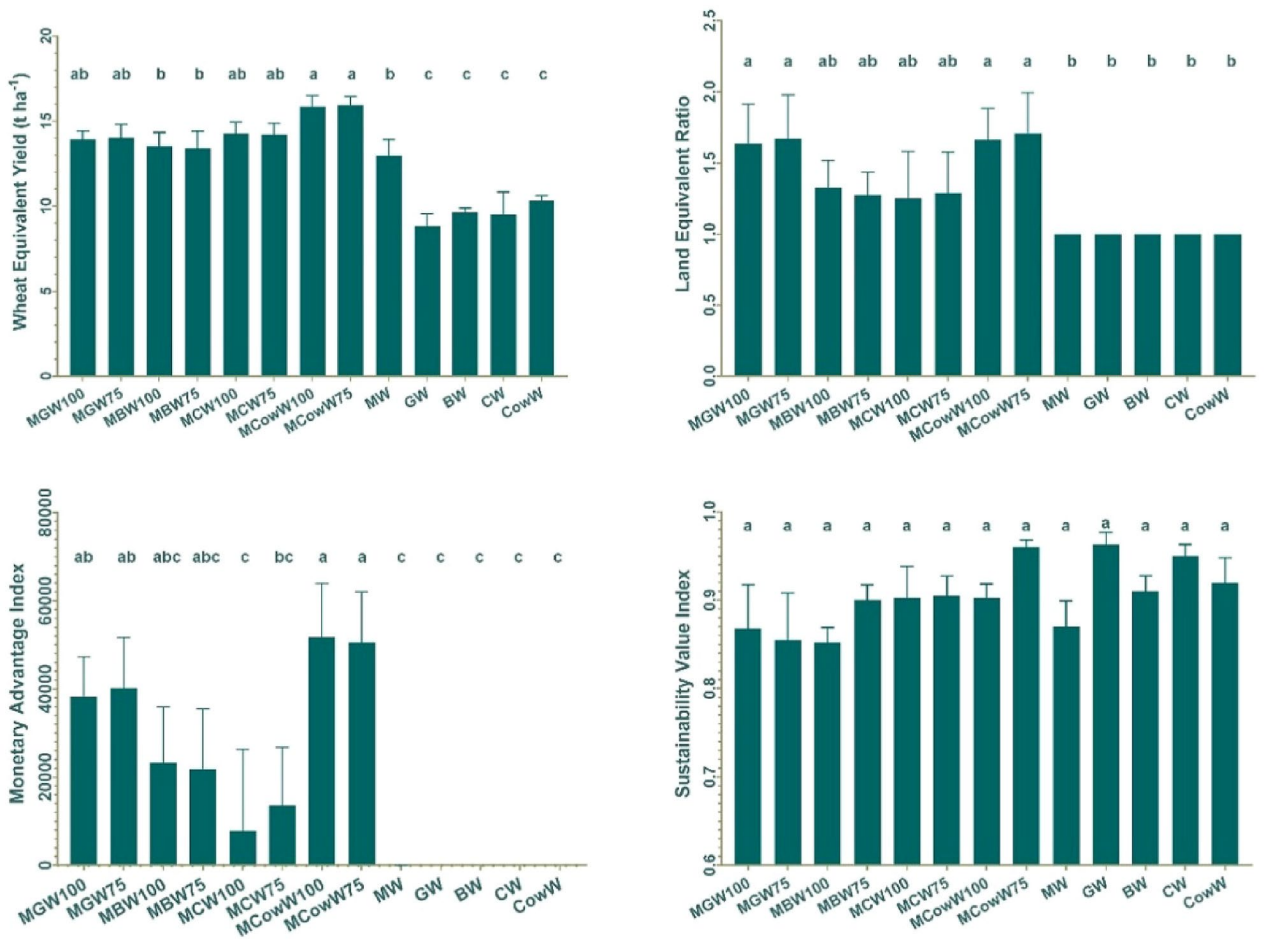
Effect of intercropping of legumes with Maize on competition indices.

System productivity was measured in terms of WEY and it was found that, MCow75 treatment produced maximum WEY (15.9 t ha^−1^) followed by MCow100 (15.8 t ha^−1^) which were statistically similar (Fig. 2). All the intercrops recorded more WEY than the maize-wheat system. This showed that system productivity under intercrops was higher than sole maize-wheat or sole legume-wheat system.

### Land equivalent ratio (LER)

All the intercrops in this study showed more than 1.0 LER, suggesting more system productivity under intercrop conditions. Maximum LER was obtained under treatments MCow75 (1.71) followed by MCow100 (1.68) and the least LER of 1 was obtained under sole crops (Fig. 2).

### Monetary advantage index (MAI)

In this study, it was observed that maximum MAI was obtained by intercropping of cowpea under MCowW100 (MAI = 47,570) and MCowW75 (MAI = 45,376) treatments. Minimum MAI was recorded under MCW100 (7326). Prices of both commodities were the same in the local market for green pod sale however higher cowpea production led to maximum MAI (Fig. 2).

### Sustainability value index (SVI)

Among the studied intercrops it was observed that all the intercrops showed more SVI than the sole maize-wheat cropping system. Maximum SVI (0.96) was obtained where cowpea intercropped with maize (Fig. 2). This confirms that cereal-cereal rotations are not performing better due to fatigued soil.

### Soil health

**Figure 3:**
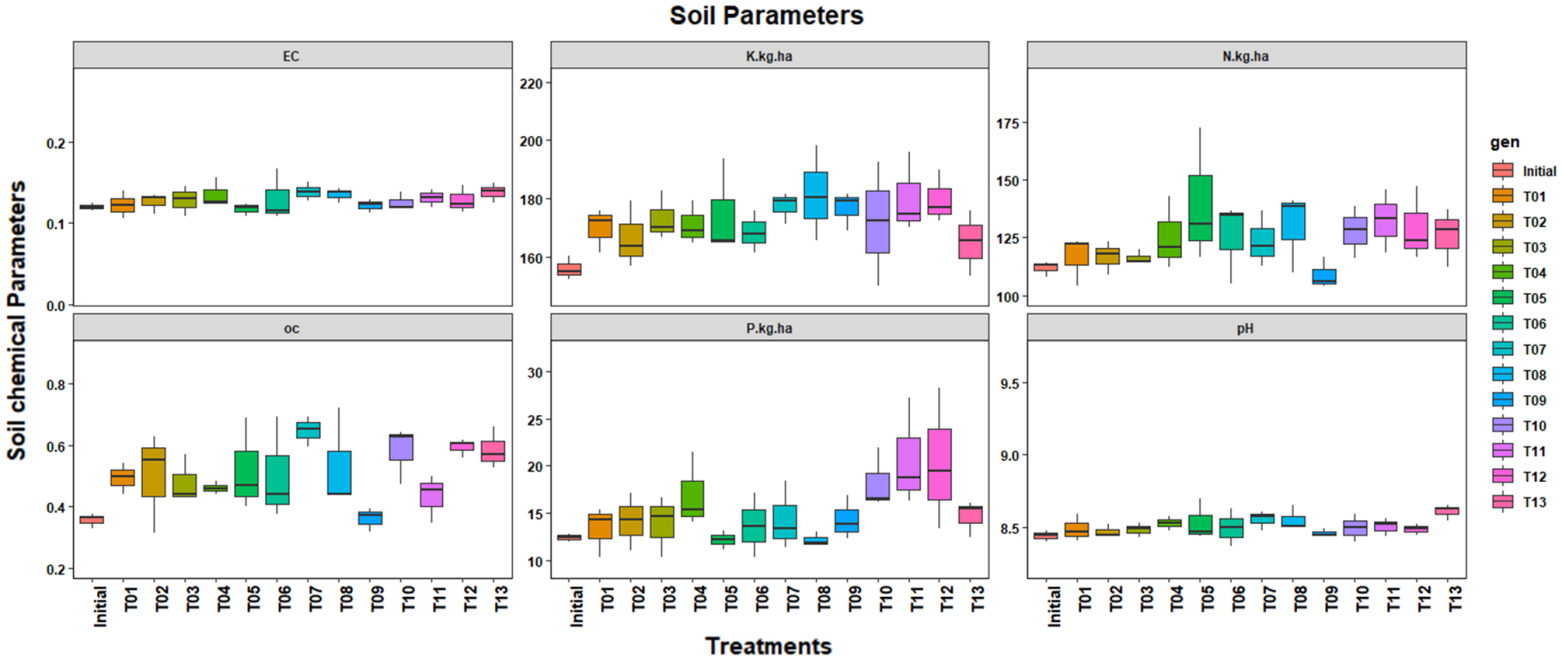
Effect of intercropping of legumes with maize on soil health measurements.

The soil profile 0–15 cm depth was analyzed for pH, EC, OC, available N, P, and K after completion of experiment and was compared with pre-experiment level. Tremendous increase in organic carbon (29.4–81.65%), available N (3.89–25.20%), available P (7.71–36.89%), and available K (6.8–16.38%) were recorded for the legume + maize intercropping treatments as compared to the initial level (Fig. 3). Cowpea intercrops with maize (MCowW) relatively enhanced organic carbon and K compared to other treatments.

### Economics

**Figure 4:**
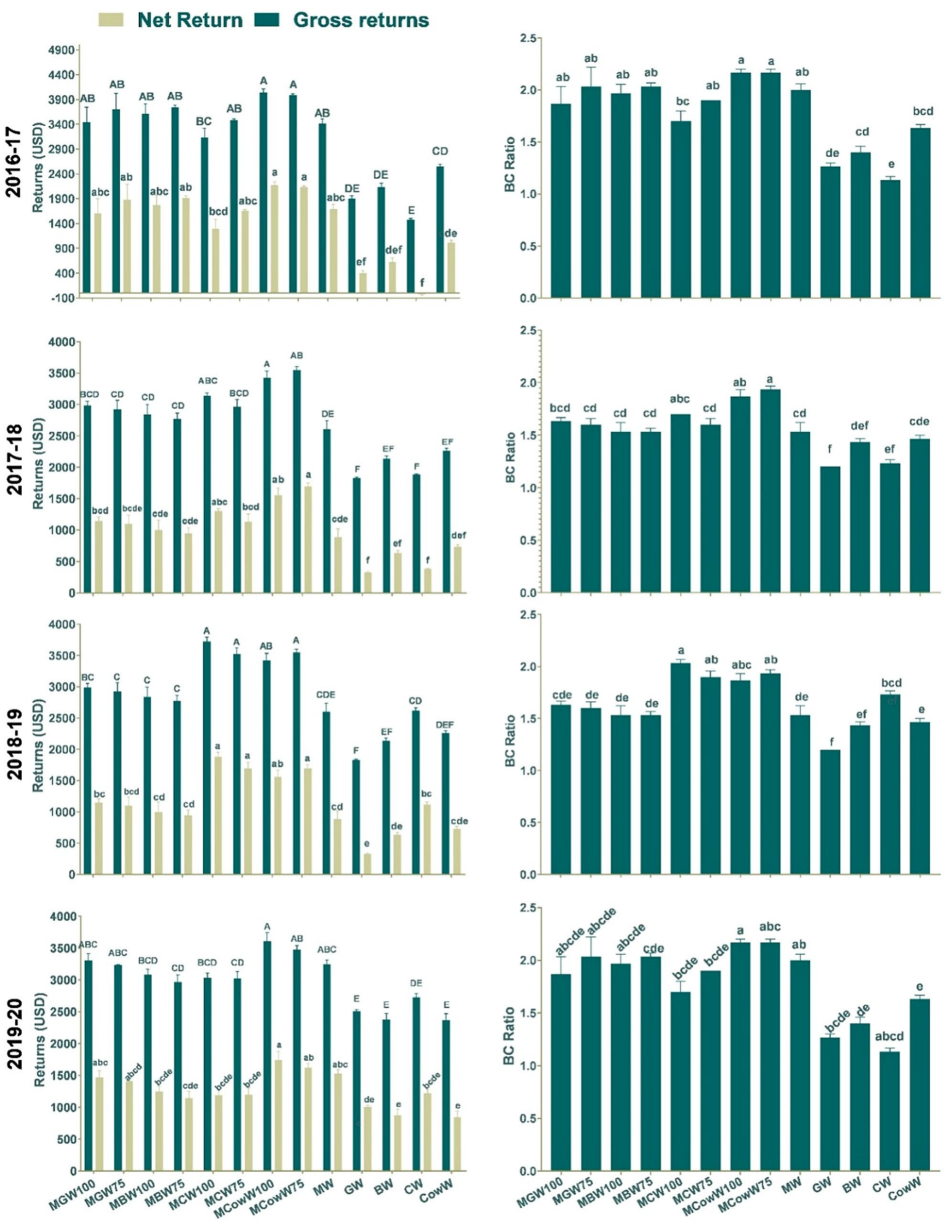
Returns (Gross and Net) and B:C ratio obtained under different intercropping treatments.

Gross return (3641 $ha^−1^), the net return (1789 $ha^−1^), and B:C ratio (1.94) was maximum under MCowW75 (Fig. 4). All the intercrops exhibited higher gross return, net return and B:C ration than sole maize-wheat. Higher B:C ratio indicated that almost double income per $ investment in this intercropping system. This confirms the hypothesis that cereal–cereal rotations are showing fatigue and it is time to utilize improved agronomic intensification strategies for generating higher returns in an environment-friendly way.

## Discussion

Maize + legume intercrop is beneficial by enabling better radiation use^5^, nutrient partitioning between component crops^6^, complementarity, and competitiveness^30^. In this study, the saving of 25% N in wheat when the legume was planted as an intercrop with maize in the previous season was the major finding. The addition of N by the pulses through biological nitrogen fixation (BNF) and enhanced carbon sequestration in the soil might be the reason for obtaining the equal yield between normal N and − 25% N treatments in wheat. Probably reduced N supply was compensated by the legume intercropping with maize leading to at par wheat yield when 25% lesser N was applied^31^. The BNF fixed N transferred to the succeeding crop was also reported earlier^32^ and further, Zao et al.^33^ observed that the favourable effects are more pronounced when soil N levels were low. Sharma and Behra^34^ reported that the N saving in the subsequent crop can also be attributed to N added through legume residues incorporation which varied from 11.5–38.5 kg ha^−1^.

Higher system productivity indicated by greater WEY in treatments containing legume as an intercrop with maize followed by wheat compared to sole maize-wheat or sole legume-wheat was observed in this study. The relative advantage of cowpea (24.8%) and green gram (16.1–29.9%) intercropped with maize as compared to the sole maize was earlier reported by Sharma and Behera^34^. Further, equivalent yields were higher under the intercropping system as compared to sole crop in a study in baby corn and legumes in eastern India^35^. Moreover, Dwivedi et al.^36^ envisaged that equivalent yield increases under intercropping situations besides nutrient supplementation to maize crop. Maize intensification through green gram leading to higher WEY than sole rice–wheat crop was also earlier reported^37,38^. The productivity of intercropping was higher due to additional legume crop in wide row spacing maize crop, their remunerative price, and similar wheat yield with lesser nitrogen application.

In this study, Mcow 75 exhibited the highest LER of 1.71 indicating that there is a requirement of 71% additional land area for the production of similar GY by sole crop. Recently, Xu et al.^6^ conducted a meta-analysis of 90 research studies on maize and soybean intercropping and found that LER of 1.32 ± 0.02 whereas in our study it was 1.71 suggesting in Indian conditions intercropping is a better approach than taking individual crops. These results were also supported by earlier reports^39,40^ where maize and legume intercropping produced higher LER and economic returns than sole crop. Greater risk-bearing capacity of diversified farming was reported^41^. Attainment of more crop per unit area by intercropping of cereals with legume crop leading to higher land-use efficiency was also reported^37,42,43^. Higher LER was reported with maize + pea intercropping^44^ and maize + cowpea^9^ than sole maize crop. In intercropping situations, MAI was more helpful in assessing the system productivity and its economic benefits^36^.

A higher sustainability value index plays an important role in maximizing the profit on a sustainable basis. MCowW 75 recorded higher SVI (0.96) and all the intercrop combinations showed more SVI than the sole maize-wheat system. These findings were supported by earlier research reports^45^ where inclusion of the green gram with rice–wheat produced a higher SVI of 0.92.

Legumes intercropped with maize increased the organic carbon (27.61–79.13%) and available N (6.7–28.59%) compared to MW treatment. In line with our observations, Lin et al.^46^ also reported that green gram, soybean, and peanut intercropped with maize showed more than double soil N as compared to the sole crop. Similarly, Hödtke et al.^47^ observed that soil organic matter in the top layer of soil increased significantly and Cong et al.^48^ found soil organic carbon (4 ± 1%), soil organic N (11 ± 1%), and 23% more root biomass with legume intercrop than sole crops, indicating more activities in the underground portion. Intercropping helps in maintaining the soil N balance on the positive side and can reduce the requirement of N fertilizer by about 26% on a global scale^49^. Sharma and Behera^34^ observed that N addition through the legume residues ranged from 11.5–38.5 kg ha^−1^ in the intercropped system, which improved the productivity of wheat. Thus, it can be said that intercropping of legumes with maize increased the soil fertility, which was utilized by the succeeding crop. Substantial reduction in dependency on external N fertilizer through the legume intercropping also has environmental benefits like reduction in transport cost, drudgery, fuel, and environmental pollution including global warming (one liter diesel burnt produce 2.6 kg CO_2_)^50^. According to reports^51,52^ 1.2% of the total global primary energy is used for chemical production of N used as fertilizers through an energy-intensive chemical process. The saved N through maize-pulse intercropping indirectly helps in reducing energy consumption for N fertilizer synthesis which otherwise would be available for alternate uses. Reduction in excess N usage would further help in maintaining water and air quality^53^.

Intercropping of the legume with maize produced higher economic returns and B:C ratio than in MW treatment. Yigezu et al.^54^ reported that legume-based rotations have clear economic advantages (48%) over cereal monocropping and this was supported by several other workers^34,39^. Additionally, increased acreage under pulse and resulting production shall lead to cheaper availability of protein. Furthermore, the availability of cheaper pulses to poorer sections of society will enhance overall health and wellbeing. The results of this study have direct global implications in countries mainly dependent on maize-based cropping systems such as China, the USA, Brazil, India, Argentina, Indonesia, South Africa, and Ethiopia^13^. These maize-producing regions worldwide can become sustainable in maize production in an environment-friendly way by the practice of maize-pulse intercropping. Excessive N usage in agricultural production and resultant environmental contamination have been reported globally^55^ in general and specifically in maize-based cropping systems of the USA^56,57^, China^58^, Pakistan^59,60^, and Brazil^61^ which could be substantially reducing their dependency on externally applied N through the practice of maize + pulse intercropping.

## Conclusions

Based on 4 years of experiments, it was established that intercropping of legumes enhanced the LER, WEY, SVI, MAI as compared to MW treatment. MCowW75 attained LER of 1.71, suggesting 71% more area is needed by the sole crop to produce the same yield. Similarly, MCowW75 recorded SVI of up to 0.96, indicating higher system productivity and it produced maximum gross return ($3641 ha^−1^), net return ($1789 ha^−1^), and B:C ratio (1.94). In this study intercropping indices have shown the advantage, economy on the positive side, and soil health increased to a great extent. Growing intercrop with legumes increased the organic carbon (27.61–79.13%) and available N (6.7–28.59%) over to MW treatment. In this article, we ummarized that maize + legume intercropping was beneficial than monoculture and it saved 25% N in succeeding wheat crop. Future research may focus on the cropping system approach rather than a single crop. From a cropping system perspective, it may be concluded that intercropping with legumes can save a significant quantity of N which in turn reduces the cost of cultivation and can enhance soil health parameters.
